# Sequencing of 6.7 Mb of the melon genome using a BAC pooling strategy

**DOI:** 10.1186/1471-2229-10-246

**Published:** 2010-11-12

**Authors:** Víctor M González, Andrej Benjak, Elizabeth Marie Hénaff, Gisela Mir, Josep M Casacuberta, Jordi Garcia-Mas, Pere Puigdomènech

**Affiliations:** 1 Molecular Genetics Department, Center for Research in Agricultural Genomics CRAG (CSIC-IRTA-UAB), Jordi Girona, 18-26, 08034 Barcelona, Spain; 2 IRTA, Center for Research in Agricultural Genomics CRAG (CSIC-IRTA-UAB), Carretera de Cabrils Km 2, 08348 (Barcelona), Spain

## Abstract

**Background:**

*Cucumis melo *(melon) belongs to the Cucurbitaceae family, whose economic importance among horticulture crops is second only to Solanaceae. Melon has a high intra-specific genetic variation, morphologic diversity and a small genome size (454 Mb), which make it suitable for a great variety of molecular and genetic studies. A number of genetic and genomic resources have already been developed, such as several genetic maps, BAC genomic libraries, a BAC-based physical map and EST collections. Sequence information would be invaluable to complete the picture of the melon genomic landscape, furthering our understanding of this species' evolution from its relatives and providing an important genetic tool. However, to this day there is little sequence data available, only a few melon genes and genomic regions are deposited in public databases. The development of massively parallel sequencing methods allows envisaging new strategies to obtain long fragments of genomic sequence at higher speed and lower cost than previous Sanger-based methods.

**Results:**

In order to gain insight into the structure of a significant portion of the melon genome we set out to perform massive sequencing of pools of BAC clones. For this, a set of 57 BAC clones from a double haploid line was sequenced in two pools with the 454 system using both shotgun and paired-end approaches. The final assembly consists of an estimated 95% of the actual size of the melon BAC clones, with most likely complete sequences for 50 of the BACs, and a total sequence coverage of 39x. The accuracy of the assembly was assessed by comparing the previously available Sanger sequence of one of the BACs against its 454 sequence, and the polymorphisms found involved only 1.7 differences every 10,000 bp that were localized in 15 homopolymeric regions and two dinucleotide tandem repeats. Overall, the study provides approximately 6.7 Mb or 1.5% of the melon genome. The analysis of this new data has allowed us to gain further insight into characteristics of the melon genome such as gene density, average protein length, or microsatellite and transposon content. The annotation of the BAC sequences revealed a high degree of collinearity and protein sequence identity between melon and its close relative *Cucumis sativus *(cucumber). Transposon content analysis of the syntenic regions suggests that transposition activity after the split of both cucurbit species has been low in cucumber but very high in melon.

**Conclusions:**

The results presented here show that the strategy followed, which combines shotgun and BAC-end sequencing together with anchored marker information, is an excellent method for sequencing specific genomic regions, especially from relatively compact genomes such as that of melon. However, in agreement with other results, this map-based, BAC approach is confirmed to be an expensive way of sequencing a whole plant genome. Our results also provide a partial description of the melon genome's structure. Namely, our analysis shows that the melon genome is highly collinear with the smaller one of cucumber, the size difference being mainly due to the expansion of intergenic regions and proliferation of transposable elements.

## Background

During recent years an important effort has been made to increase the tools available for the genomic analysis of major plant crop species. Since the first genome sequence available of *Arabidopsis thaliana *[1], several others have been published. They include model plants such as *Brachypodium *[2] but, increasingly, species that have been chosen for their importance in agriculture. For example the rice [3], maize [4], sorghum [5] or soybean [6] genomes are complex but the wealth of genetic information matches their economic interest. Consequently, for both scientific and economic reasons an increasing number of plant genomes are being analyzed, providing important resources useful for their biological study and breeding.

Several species of interest from both scientific and economic perspectives are of the Cucurbitaceae family. These include melon, cucumber, watermelon and squashes, all of which have been the object of biological and agricultural interest for centuries. In recent years various molecular tools have been established. For instance, the first assembly of the cucumber genome [7], as well as an increasing number of genetic and genomic resources developed for melon, a diploid species with a relatively compact (around 454 Mb [8]) genome [9]. These include tools such as a collection of more than 129,000 ESTs [10,11], BAC libraries [12,13], oligo-based microarrays [14,15], TILLING and EcoTILLING platforms [16,17], a set of near isogenic lines (NILs) [18] and several melon genetic maps [11,19-25]. Recently, we have built a physical map with 0.9x genomic coverage using both a BAC library and a genetic map previously developed in our laboratories [http://melonomics.upv.es/public_files, [26]], the first report of such a genomic resource of a Cucurbitaceae species so far. This physical map has also been integrated with the genetic map by anchoring a number of physical contigs (representing 12% of the melon genome) to 175 known genetic markers. These tools have been useful in the study of interesting agronomical traits such as virus or fungi resistance [27,28], sex determination [29,30] or the control of ripening [31,32]. These results demonstrate that molecular genetic approaches can successfully be used in melon to address basic questions of biological or agronomic relevance.

More extensive sequence information would be invaluable to complete the picture of the melon genomic landscape. Indeed, the sequences of only a few selected genomic regions have been published, totaling no more than 500 kb [29,33-35] and as of May 2010 no more than 173 melon genes can be found in GenBank [11], although a collection of ESTs probably representing more than 70% of the transcriptome is currently available [11]. The sequencing of the Sorghum genome has shown the feasibility of sequencing a plant genome larger than that of melon (730 Mb) using a Sanger-based whole genome shotgun approach [5]. However, the development of new massively parallel sequencing technologies allows envisaging a complete sequencing of the species at higher speed and at lower cost than previous Sanger-based methods. To this end, both whole genome sequencing approaches as well as map-based, BAC-to-BAC strategies have been proposed to sequence plant genomes [36,37].

A small number of research projects involving 454 sequencing of BAC clones have currently been published. In a pioneering study aimed at analyzing how 454 technology would perform on template derived from large genomes rich in repetitive content, four barley BAC clones 102-120 kb long, two of which had been previously sequenced using Sanger technology, were sequenced using 454 [38]. The results showed that gene-containing regions could efficiently and accurately be assembled into contigs, even at read coverages as low as x10.

In a later work eight BACs belonging to a minimum tiling path covering *ca*. 1 Mb of the Atlantic salmon genome were sequenced using 454 technology, the first published use of paired-end reads for *de novo *sequence assembly [39]. This study demonstrated that although the inclusion of paired-end reads greatly improved sequence assembly, there remained a significant number of gaps when compared to Sanger-generated sequencing data. Thus the authors concluded that, when it comes to *de novo *sequencing complex genomes, 454 sequencing should be restricted, at least for the time being, to establishing a set of ordered sequenced contigs.

Although these studies show that 454 sequencing can be used to assemble gene-containing regions from genomic sequences using a BAC-to-BAC approach, the cost of 454 sequencing individual BACs has led to consider pooling individual clones as a means to increase throughput and reduce the cost of genome sequencing. In one published study, 166 BACs totalling 20 Mb were divided into six pools of overlapping BACs, aided by paired-end sequencing. These were then used to 454-sequence a minimum tiling path which covered an entire chromosome arm from *Oryza barthii *[37]. The report shows that pooling BACs does not increase the complexity to a degree that makes assembly impossible, what makes this approach a feasible strategy for reducing the cost of BAC sequencing. In another work 91 barley BAC clones, pooled by sets of 12 or 24, were sequenced using 454 technology [40]. The introduction of short sequence tags to fragmented BAC DNA prior to pooling and sequencing helped to resolve the assembly of multiplex sequencing data by establishing relationships between BAC clones and sequence reads, reducing sample complexity.

Here we present a pilot project aiming to sequence two pools of 35 and 23 melon BACs using the 454 system and a combination of shotgun and paired-end sequencing. The goal of the study was twofold: obtain sequence data for a significant proportion of the melon genome and thus insight into its structure, and test the strategy of massively sequencing pools of BACs. The results obtained allow an accuracy assessment of 454 sequence and assembly data as compared with sequence data produced using classical Sanger technology. Overall, the study provides approximately 7 Mb or 1.5% of the melon genome as a first step towards the complete sequence. The analysis of this data has provided insight into characteristics of the melon genome such as gene density, transposon content and synteny with cucumber.

## Results and discussion

### Selection of BAC clones for pooling and sequencing

Two pools of DNA prepared from BACs were sequenced using the 454 pyrosequencing method. These BACs had been produced from DNA of the double haploid line PIT92 obtained from the cross of PI 161375 and T111 as described in [12].

**Figure 1:**
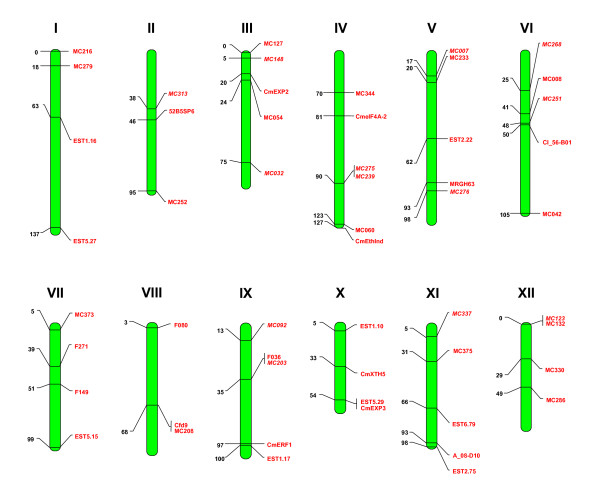
**Distribution of the genetic markers used to anchor the sequenced BACs to the *C. melo *genetic map**. Linkage groups are numbered according to the *C. melo *map of Deleu *et al. *[24]. Map distances are indicated on the left in cM. Markers in italics have been placed in an approximate position from Oliver *et al. *[21].

A set of 178 genetic markers selected from previous versions of the PI 161375 × T111 melon genetic map (mainly RFLPs [21] and SNPs [24,31,41,42]) were used to anchor 845 BAC clones from our genomic library to the genetic map [26]. Of these, a batch of 32 BACs anchored to genetic markers distributed throughout the genome (See Figure 1) were chosen for 454-sequencing. In order to test the quality of the sequencing and assembly procedures, one previously Sanger-sequenced BAC (Cm13_J04, Acc. No. EF657230.1) was selected from the MRGH63 contig constructed on the basis of BAC end information [12,35]. We also added to the pool BACs Cm43_H20 and Cm14_M22 of this contig that are known to overlap with the former (Additional file 1 Figure S1). In all, this first pool of BAC clones consists of 35 BACs mapping to 33 different loci.

A second batch of 20 BACs anchored to genetic markers distributed throughout the genome but different from those corresponding to the first set of 35 BACs was also chosen for 454-sequencing (see Figure 1). Three additional BACs were included in this second pool: the above-mentioned BAC Cm43_H20, and two randomly chosen BAC clones not linked to any known genetic marker (BACs Cm21_I02 and Cm12_I23). In all, the second pool consists of 23 BACs mapping to at least 21 different genetic loci.

In all, the selected two sets of BACs represent an estimated 7.5 Mb of the melon genome, based on BAC library average insert size. The complete list of selected BAC clones, together with their corresponding genetic markers can be found in Table 1. Information regarding genetic map position, marker type and references of the genetic markers can be found in the Additional File 2 Table S1.

**Table 1:** Correspondence between sequenced BAC clones, genetic markers and assembled contigs/scaffolds.*

			Scaffold					
			
Linkage group	**Marker name**^**a**^	BAC name	Name	GenBank ID	Length (bp)	Stretches of Ns		BAC-ends **Found**^**b**^
							
						**No**.	Length (bp)	
								

I	MC216	Cm57_M11^2^	Contig311	HM854822	626	0	0	0
I	MC279	Cm31_J02^1^	Scaffold00087	HM854819	126,619	3	1,334	2
I	EST1.16	Cm33_F23^2^	Scaffold00078	HM854813	113,787	11	9,016	2
I	EST5.27	Cm43_O21^1^	Scaffold00052a	HM854797	131,697	10	3,452	2
II	MC313	Cm05_B01^2^	Scaffold0006	HM854766	126,054	4	4,978	2
II	52B5SP6	Cm52_B05^1^	Scaffold52B05	HM854760	138,922	38	29,090	2
II	MC252	Cm46_G13^1^	Scaffold0009	HM854768	151,031	2	529	2
III	MC127	Cm05_P10^2^	Scaffold05P10	HM854751	114,263	7	5,100	1
III	MC148	Cm45_K10^2^	Scaffold00035	HM854788	105,652	2	630	2
III	CmEXP2	Cm24_H21^1^	Scaffold0003^3^	HM854763	86,310	3	1,125	2
III	MC054	Cm52_C09^1^	Scaffold00024b	HM854780	61,053	2	666	1
III	MC032	Cm55_F19^1^	Scaffold55F19	HM854755	110,853	11	9,792	2
IV	MC344	Cm33_M05^2^	Scaffold00077	HM854812	148,622	6	2,636	2
IV	CmelF4A-2	Cm59_B11^1^	Scaffold59B11	HM854756	100,000	5	5,558	2
IV	MC275	Cm11_I12^1^	Scaffold11I12	HM854757	110,000	9	6,210	2
IV	MC239	Cm06_A03^1^	Scaffold00012a	HM854770	75,205	0	0	1
IV	MC060	Cm46_O06^2^	Scaffold00041^3^	HM854791	103,741	2	668	2
IV	CmEthInd	Cm14_C18^1^	Scaffold0001	HM854762	108,322	1	478	2
V	MC007	Cm52_M23^2^	Scaffold00070	HM854810	112,968	2	567	2
V	MC233	Cm24_G05^1^	Scaffold00017	HM854775	82,645	1	247	2
		
			Contig00219	HM854821	810	0	0	0
V	EST2.22	Cm46_I24^1^	Scaffold00044	HM854793	12,974	4	4,832	1
			Scaffold00071	HM854811	20,426	7	9,835	1
		
V	MRGH63	Contig MRGH63:	ScaffoldMRGH63	HM854749	302,015	9	4,457	
		Cm13_J04^1,4^						2
		Cm14_M22^1^						2
		Cm43_H20^1,2^						2
		
V	MC276	Cm01_N3^1^	Scaffold00015	HM854773	180,444	5	2,607	1
VI	MC268	Cm02_C04^2^	Scaffold00031	HM854785	105,693	4	1,877	2
VI	MC008	Cm31_G08^2^	Scaffold00033	HM854786	109,145	3	764	2
VI	MC251	Cm02_K14^1^	Scaffold00058	HM854801	121,212	8	2,788	2
VI	CI_56-B01	Cm27_F03^1^	Scaffold27F03	HM854758	96,265	1	506	2
VI	MC042	Cm20_H14^1^	Scaffold00018	HM854776	96,294	3	891	2
VII	MC373	Cm55_C15^1^	Scaffold00057	HM854800	98,578	1	316	2
VII	F271	Cm45_K01^1^	Scaffold45K01	HM854759	100,000	11	7,803	2
VII	F149	Cm47_C02^2^	-	-	-	-	-	-
VII	EST5.15	Cm47_A05^1^	Scaffold0004	HM854764	101,589	3	1,515	2
VIII	F080	Cm22_K19^1^	Scaffold00081	HM854815	99,638	6	2,137	2
VIII	Cfd9	Cm06_D16^1^	Scaffold00025	HM854781	102,876	13	10,731	1
VIII	MC208	Cm19_K17^2^	Scaffold00023	HM854779	125,428	1	242	2
IX	MC092	Cm24_H03^2^	Scaffold24H03	HM854823	106,131	3	1,282	2
IX	F036	Cm34_G20^1^	Scaffold00069	HM854809	125,129	2	1,825	2
IX	MC203	Cm54_J04^2^	Scaffold54J04	HM854753	100,000	6	13,775	2
IX	CmERF1	Cm54_I13^1^	Scaffold54I13	HM854824	94,153	3	1,036	2
IX	EST1.17	Cm10_D04^1^	Scaffold10D04	HM854761	154,039	45	28,530	2
X	EST1.10	Cm03_A21^1^	Scaffold00079	HM854814	126,557	2	482	2
X	CmXTH5	Cm41_H09^1^	Scaffold0005	HM854765	136,275	2	696	2
X	EST5.29	Cm19_G01^2^	Scaffold00013	HM854771	100,283	14	8,175	2
X	CmEXP3	Cm54_E01^2^	Scaffold54E01	HM854754	100,000	18	13,832	2
XI	MC337	Cm12_F09^2^	Scaffold00028	HM854783	118,830	3	1,840	2
XI	MC375	Cm03_C12^1^	Scaffold00014	HM854772	128,906	13	9,538	1
XI	EST6.79	Cm59_N09^1^	Scaffold00085	HM854817	102,799	1	338	1
XI	A_08-D10	Cm24_I03^2^	Scaffold24I03	HM854752	121,276	16	8,734	1
XI	EST2.75	Cm33_O17^1^	Scaffold00051	HM854796	123,309	3	1,296	2
XII	MC123	Cm59_C10^2^	Scaffold59C10	HM854750	10,343	3	1,861	0
XII	MC132	Cm03_I02^1^	Scaffold00086	HM854818	79,495	4	3,249	1
XII	MC330	Cm09_A17^1^	Scaffold00034	HM854787	96,336	1	271	2
XII	MC286	Cm05_O10^2^	Scaffold00020	HM854778	142,670	8	2,708	2
-	-	Cm21_I08^2^	Scaffold00061	HM854803	146,020	12	5,169	2
-	-	Cm12_I23^2^	Scaffold00010	HM854769	114,336	9	8,517	2

*Additional information regarding sequence and annotation characteristics of the assembled sequence can be found in the Additional file 3 Table S2

^a^Genetic marker information can be found in the Additional file 2 Table S1

^b^One (1), both (2) or none (0) BAC-ends found on the scaffold/contig sequence

^1^First pool of BACs

^2^Second pool of BACs

^3^Marker sequence not found. Scaffold assignment based on information derived from the *C. melo *physical map http://melonomics.upv.es/static/files/public/physical_map/ and BAC-end information

^4^Sequenced previously by Shotgun-Sanger [35], Acc. No. EF657230

### Sequencing and assembly

Both shotgun and 3 kb paired-end libraries were constructed for each pool of BACs and the sequencing was carried out independently as described in the Methods section. A summary with the details of the different 454 runs, including number of reads, total length and average read size can be found in Table 2. In total, over one million reads representing 274 Mb of sequence from the 35 BACs pool and over 400,000 reads totaling 105 Mb from the 23 BACs pool were produced. The raw data (sff files) have been deposited in the SRA archive of the NCBI under the accession number SRA024701.1.

**Table 2:** Details of the 454 FLX runs from which sequence data were obtained.

Pool	Sequencing plate regions	Library type	No. of reads	No. of Paired-end reads	Total length (bp)	Average read size (bp)
	
						
**35 BACs**						
	**2/2**^**a**^	Shotgun	445,232	-	110,498,601	248
						
	2/4	Paired end	89,392	3,152	23,214,413	260
	2/2	Paired end	557,452	126,681	139,772,537	251

**23 BACs**						
	2/2	Shotgun	261,304	-	64,679,158	247
	3/8	Paired end	155,166	56,990	40,110,640	259

^a^Includes 8,046 reads obtained from the titration process of the samples as well as 20,627 reads from a 1/2 region that was poorly sequenced

A global assembly of all reads from both BAC pools was performed as described in the Methods section. In addition, two independent assemblies were performed using reads from each pool. The reduced complexity in the separate assemblies of individual pools of BACs would suggest a more accurate assembly. Indeed, the number of contigs slightly increases and their size decreases in the global assembly, but overall, the result of the global assembly resembles the results from the assemblies of the individual BAC pools, except for a few cases. For example, in the case of the BAC Cm54_I13, we obtained a single scaffold in the 35 BACs pool assembly corresponding to two scaffolds from the global assembly. What separates the two scaffolds (when aligned to the single one) is a 273 bp gap flanked by several TA motives. On the other hand, scaffold00040 from the global assembly contained 631 additional nucleotides and a 522 bp long gap flanked by AT repeats at one of its extremes compared to its counterpart scaffold from the 23 BACs pool assembly. As we do not have a reference genome, we considered the larger scaffold as reference. A detailed summary of the whole process with the metrics of the three assemblies can be found in Table 3. Based on this information, we conclude that for assembling a modest number of BACs it is not worth separating them in smaller pools (increasing the sequencing costs), and if reduction of complexity is imperative (when dealing with very repetitive genomes, for example) then the extreme approach could be considered and barcode each BAC.

**Table 3:** Metrics for BAC assemblies and final results after manual correction.*

	35 BACs	23 BACs	Global assembly 57 BACs (two pools together)	Manual correction
**No. of contigs**^**a**^	514	247	797	-
**No. of bases in contigs**	3,936,343	2,325,066	6,127,262	-
**Average contig size (bp)**	7,658	9,413	7,687	-
**N50 contig size (bp)**	32,583	32,458	30,630	-
**Largest contig size (bp)**	117,242	112,451	123,360	-
**Q40 plus bases**	99.5%	99.5%	99.5%	-

**No. of scaffolds**	58	32	87	73
**No. of scaffolds larger than 20 kb**	41	25	62	57
**No. of bases in scaffolds**	4,040,161	2,307,575	6,206,490	6,340,685
**Average scaffold size**	69,657	72,111	71,338	86,882
**N50 scaffold size**	107,196	113,599	107,604	113,787
**Largest scaffold size**	222,620	200,453	212,424	303,725^b^
**No. of unscaffolded contigs**^**c**^	479	234	798	744
**No. of bases in unscaff. contigs**	224,871	121,734	417,982	382,726
**Average unscaff. contig size**	469	520	524	514
**Coverage**	x46	x25	x39	x39

*Reads from all 57 BACs were processed together in one assembly run. Additional assemblies of each BAC pool were independently done and served for comparison purposes and to manually correct some scaffolds in the global assembly

^a^Only contigs larger than 500 bp

^b^Two previously published BACs were included in this scaffold (see Methods section and Additional file 1 Figure S1)

^c^Contigs larger than 100 bp

The assignment of contigs and scaffolds to BACs was performed using anchored genetic markers and BAC-end sequences as described in the Methods section. Also, the information from the *C. melo *FPC physical map [26] together with BAC-end sequences from some BAC clones in FPC contigs allowed us to manually edit two scaffolds of the final assembly. The physical map was also useful in assigning BACs Cm21_I08 and Cm12_I23 to their corresponding scaffolds, as no genetic markers correspond to these BACs. Finally, the previously Sanger-sequenced BAC Cm60_K17 (Acc. No.: AF499727.1, [12]) was added to the alignment of the sequenced BACs from the MRGH63 contig in order to extend the sequence used for subsequent analysis (see Additional file 1 Figure S1).

The final assembly consists of 73 scaffolds totaling 6.3 Mb, 73% of which are longer than 60 kb, with average scaffold size 86.8 kb and the largest scaffold 304 kb long; also, 744 unscaffolded contigs totaling 382 kb of sequence remain (Table 3). The sequence coverage of the final assembly is 39x, calculated as the ratio between the total length of the sequence reads and the assembly sequence length. Paired-end reads are used in the process of sequence assembly to join contigs (formed by read alignments) in structures called scaffolds, which represent sorted and correctly orientated contigs that are separated by gaps which sizes are estimated based on the average paired-end size (see, for example, [39]). The N50 contig size of our assembly was rather small (30.6 kb) compared to the N50 scaffold size (107.6 kb). This result confirms the importance of paired-ends when it comes to assembling a complex genome using 454 sequences.

Regarding the assignment of sequences to particular BACs, BAC Cm47_C02 could not be assigned to any scaffold or contig and BAC Cm46_I24 was assigned to a small contig of less than 1 kb using the genetic marker sequence information, and to another two small scaffolds using both BAC-end sequences. All other BACs were assigned to a unique scaffold or contig, two of which were smaller than 15 kb, another five in the 60-90 kb range while the rest was over 90 kb long (Table 1).

The search for BAC ends in the final set of contigs and scaffolds suggests that at least 42 scaffolds cover the complete sequence of 44 BACs (including the three BACs belonging to the scaffold MRGH63). An average of seven stretches of Ns (produced as a result of contig scaffolding) was found per scaffold and the total length of all Ns accounts for 4.8% of the final assembly length (see Additional file 3 Table S2). Nine additional scaffolds assigned to as many BAC clones were found to contain only one BAC border each; however, six of these scaffolds were bigger than 100 kb, and so they probably represent complete BAC sequences but for small deletions at their borders, while the rest measured between 60 and 80 kb and could represent a significant proportion of their correspondent BAC sequences. Finally, BAC borders were absent from two BAC sequences (corresponding to BACs Cm57_M11 and Cm59_C10), both smaller than 11 kb and therefore most likely incomplete.

As a summary, of a total of 57 pooled BACs, most likely complete sequences were produced for 50 BAC clones, three were incomplete but in the range of 60-80 kb and four BACs were attributed very limited sequence information. As the assignment was performed using a small amount of sequence information, namely the marker and BAC-end sequences (not available for all BACs), any sequence shorter than the full BAC insert size has few chances of being assigned to any particular BAC. This is obvious for the BAC Cm46_I24 where with each BAC-end sequence and the marker sequence we assign three rather small sequences (Additional file 3 Table S2). In our results, a total of 374 kb distributed in 20 contigs/scaffolds longer than 2,000 bp remained unassigned after the final assembly and could account for most of the sequence of those four problematic BACs. All markers but one (F149), and all available BAC-end sequences but three, matched against a contig or scaffold. The nucleotide sequences of contigs and scaffolds assigned to BACs as well as of those unassigned assembly sequences larger than 2 kb have been deposited in the GenBank database and their accession numbers can be found in the Additional file 3 Table S2.

The number of gaps per Mb (61) and the estimated amount of missed sequence in our main assembly (5%) are lower than those values from the above-mentioned studies using 454 sequencing of BAC clones [37-39], a fact most probably due to the absence of paired-end sequencing in [38,40], to the short reads that were being produced at the erlier stages of 454 technology (100 bp on average) [38], to the complexity of the barley and salmon genomes as compared with melon's [38-40], and to the higher amount of assembled sequence in the case of *O. barthii *[37]. In summary, although using shotgun and paired-end libraries of pooled BACs remains a costly proposition for sequencing a whole genome, it is well adapted to certain situations. Indeed, our results show that it is a feasible and cost-efficient strategy for sequencing particular regions of interest of relatively compact genomes like that of melon. This approach would also be useful in genome walking strategies for gene cloning, or resolving a particular region where a physical map is available.

### Sequence accuracy assessment

The quality of the final assembly was assessed by comparing the sequence from scaffold MRGH63 corresponding to BAC Cm13_J04 (Additional file 1 Figure S1) against the 99 kb-long sequence of the same BAC previously obtained using a shotgun-Sanger approach [35]. Table 4 shows the differences between the Sanger and 454 sequences. Apart from five small stretches of Ns representing 3.6% of the BAC length, the only other discrepancies are 15 homopolymeric regions and two dinucleotide tandem repeats. The differences in homopolymeric regions were found in 15 of the 26 mononucleotide repeats longer than 11 nt, and in all cases but one the 454 repeat resulted to be one to three nucleotides shorter than the Sanger sequence. It is interesting to note that no differences were found in the 896 mononucleotide repeats shorter than 11 nt. The discrepancies in dinucleotide tandem repeats affected two (CT)_15 _and (GA)_21 _microsatellites.

**Table 4:** Differences between Sanger- and 454-sequences of BAC Cm13_J04.

Length of Sanger-sequence	98,716 bp			
Stretches of Ns on 454-sequence	5	3,572 bp (3.6%)		
**Homopolymers**	**length**	**Sanger**^**1**^	**454 differences**^**2**^	
			**No.**	**Motif**
**A/T**				
	≤10	847	0	
	11	5	0	
	12	5	3	(A/T)_11_
	13	3	2	(A/T)_12_
	14	2	1	(A/T)_13_
				
	15	3	2	(A/T)_14_
			1	(A/T)_13_
				
	16	1	1	(A/T)_14_
	17	3	2	(A/T)_15_
	18	1	1	(A/T)_17_
	22	1	1	(A/T)_19_
	24	1	0	
	28	1	1	A_15_CA_13_
**C/G**				
	5-7	49	0	

**Other**	**Sanger**	**454**		
	(CT)_15_	(CT)_15_CTACTTACTTACTTACNNNNNNNC(CT)_14_		
	(GA)_21_	(GA)_21_GTAGTACGTACN_23_(GA)_6_		

^1^Number of homopolymers in the Sanger sequence

^2^Number of homopolymers in the 454 sequence showing differences with the corresponding homopolymers in the Sanger sequence

It has been already described that Sanger and 454 technologies have a generally comparable level of accuracy regarding genic regions or other single-copy sequences, homopolymeric stretches being the main source of read errors in both techniques when low copy regions are considered [37,38,43,44]. Previous reports have also shown that longer stretches of A and T are more likely to cause problem when using pyrosequencing [38]. Indeed, there is a tendency of homopolymers to be shorter in the 454 sequence than in the Sanger reads, although at least a report exists where the stretches were consistently found to be one nucleotide longer in the 454 sequences [38,43]. In summary, the polymorphisms detected between the melon 454 and Sanger sequences in a 100 kb interval involved only 1.7 differences every 10,000 bp, a figure close to previously reported values [37,38].

Besides homopolymers, repetitive DNA is known to be more problematic for 454 sequencing than for Sanger due to the shorter length of the 454 reads. Repetitive regions can be collapsed into one consensus contig causing gaps to appear in the final assembly. This may be the main reason behind the gaps accounting for an estimated loss of *ca*. 5% of melon sequence in our final assembly. Indeed, all five stretches of Ns in Cm13_J04 consensus sequence are found in two regions that contain repetitive sequences such as a transposable element and a TIR-NBS-LRR resistance gene (data not shown).

### Sequence annotation

*Ab initio *prediction of protein coding, tRNA and rRNA genes was carried out as described in the Methods chapter. The predictions were validated by homology with protein sequences at NCBI databases and with ESTs from the melon unigene v3 database at ICUGI [11]. A census of simple sequence repeats (SSRs) was also performed using the msatcommander software.

A summary of the sequence and annotation features of all 58 contigs and scaffolds longer than 20 kb, representing 6.2 Mb of genomic sequence, can be found in Table 5. As a whole, 616 protein coding genes (excluding transposons) were predicted, of which 73.2% were found to show homology with known *C. melo *ESTs. The average gene density is estimated to be 9.9 genes for each 100 kb but varies on the 2-20 range when individual scaffolds are considered; the average intron and exon length are respectively 393 bp and 238 bp and number of exons per gene is 4.9, with 46% of coding sequence being introns. Predicted proteins were 386 aa long on average. Regarding SSRs, 4,430 microsatellites were found representing 1.25% of the total sequence, about one SSR every 1.3 kb. The GC content composition was 33%, eleven tRNA genes were found in five BAC clones and no rRNA genes could be found in the analyzed sequence. Additional file 3 Table S2 contains a more detailed report of the individual characteristics of each scaffold or contig larger than 2 kb.

**Table 5:** *C. melo *BAC sequences characteristics^a^.

**Total sequence length**	6,230,040 bp
**Sequence length excluding stretches of Ns**	5,958,994 bp
**Number of predicted protein coding genes**^**b**^	616
**Number of predicted protein coding genes with homology to *C. melo *ESTs**	451 (73.2%)
**tRNA genes**	11
**Gene density**^**c**^	9.9 genes/100 kb (1.5 - 19.7, SD: 4.3)
**Average exon length**	238 bp
**Average intron length**	393 bp
**Exons per gene**	4.9 (1-29, SD: 4.4) (74% of genes ≤ 6 exons) (23% intronless)
**Average protein length**^**d**^	386 (34-2,156, SD: 268)
**Average% of introns in coding sequence**^**e**^	45.6 (4.3 - 95.5, SD: 20.6)
**GC content (%)**	33 (30.2 - 38.7, SD: 1.34)
**SSRs**^**f**^	4,430 (74,590 bp, 1.25% of total sequence) 1 SSRs/1.3 Kb
**Transposable elements**^**g**^	139

^a^From the analysis of all 57 scaffolds plus one contig longer than 20 kb

^b^Genes from transposons not counted

^c^Partial genes at BAC borders counted as 0.5 genes

^d^Transposon proteins not considered

^e ^ORFs without introns not considered

^f^Minimum repeat lengths considered: 10 bp (mononuc.), 12 bp (di- and trinuc.), 16 bp (tetranuc.), 20 bp (pentanuc.) and 24 bp (hexanuc.)

^g^See Table 6 for a more detailed analysis of transposon content

The recent publication of the *Cucumber sativus *genome sequence begs the comparison of sequence and annotation characteristics of both cucurbit species [7]. Overall, the statistics of protein-coding genes from both cucurbits are quite similar. The predictions for the cucumber genome are a gene density of 10 per 100 kb, mean protein length of 349 amino acids, average number of exons per gene, exon length and intron length of 4.8, 238 bp and 483 bp, respectively, and tRNA gene density of 2.9 per Mb. While the gene density, mean exon length and average number of exons per gene are very similar in both species, in cucumber the protein length is only slightly smaller (0.9x), and mean intron length is just 1.2 times greater.

The apparent similar gene density, together with the similarity in average protein length, number of exons and average exon and intron lengths, seems contradictory with the difference in genome size between both species. Indeed, the estimated size of the melon genome is 1.3x that of cucumber [7,9]. It has to be taken into account, however, that the cucumber gene density was calculated based on as much as 70% of the complete genomic sequence, which most probably included gene-poor regions, while the melon gene density has been estimated using BAC clones that have gene- or EST- based genetic markers and thus probably represent gene-rich regions. Therefore, it might be the case that the actual melon gene density is lower than that of cucumber, hypothesis that is supported by the analysis of syntenic regions from both genomes (see below in the "Analysis of microsynteny" section).

### Transposon content of the sequenced BACs

Transposons were annotated using sequence similarity searches with previously characterized transposons as well as by *Ab initio *methods based on transposon structural characteristics. As expected, most of the elements found belong to the retrotransposon class of mobile elements, with the *Gypsy *family being the most represented. However, the fraction of the genome these elements occupy is apparently smaller than in other genomes of similar size. Indeed, while retrotransposons account for 20% of the genomes of grapevine (504.6 Mb) and *Lotus japonicus *(472 Mb) [45,46], these elements seem to account for only 7.2% of the melon genome (454 Mb) (Table. 6). Retrotransposons are not randomly distributed in genomes and while some elements preferentially integrate in gene-rich regions (see for example [47]), others target heterochromatic regions for integration, in particular those belonging to the *Gypsy *family which are usually present at higher copy number [48]. Thus, the apparent low retrotransposon copy number could be due to the fact that heterochromatic regions are under-represented in the 1.5% fraction of the genome analyzed, which was selected to be representative of the gene-rich regions of the melon genome.

We have also found representatives of all the major families of DNA transposons, including CACTA, MULE, hAT, PIF and Helitron elements, covering in total 0.93% of the analyzed sequence (Table 6), which is consistent with what has been reported for the genomes of grapevine (1.98%) [49] and *Lotus japonicus *(0.97%) [46].

**Table 6:** Transposon content in the *C. melo *sequenced BACs.^a^

Family	Copies (no.)	Total lenght (bp)	Analyzed sequence (%)
**DNA transposons**			
**CACTA**	15	30,238	0.48
**hAT**	4	8,726	0.14
**MULE**	6	17,836	0.28
**PIF**	1	842	0.01
**helitron**	1	830	0.01
	
**Total**	**27**	**58,472**	**0.93**
	
**Retrotransposons**			
***Copia***	15	49,606	0.79
***Gypsy***	18	80,452	1.28
**Non-LTR**	3	9,664	0.15
**Non-classified**	77	313,326	5.0
	
**Total**	**113**	**453,048**	**7.2**

^a^From the analysis of all contigs and scaffolds longer than 2 kb

### Analysis of microsynteny

**Figure 2:**
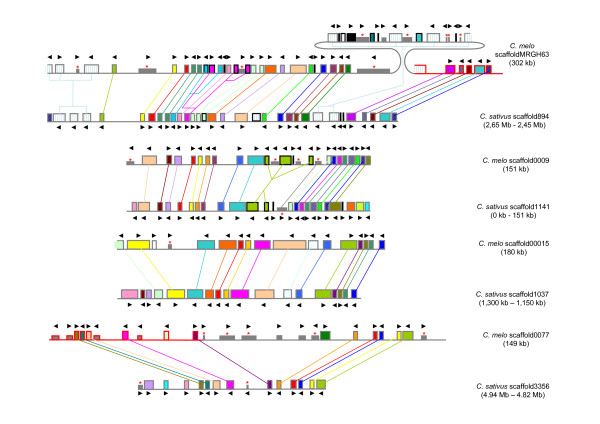
**Overview of microsynteny between four melon scaffolds and four regions in the *C. sativus *genome**. Genes are represented by square blocks. Homologous genes are illustrated with the same colour and indicated by connecting lines of the corresponding colour. *Ab initio *predicted genes with no homology to public EST or protein databases are shown in black. Transposable elements are in gray, with red asterisks as an additional mark for retrotransposons. Genes coding for NBS-LRR disease resistance proteins are represented by square block filled with blue vertical lines. Putative pseudogenes are depicted as black edge boxes. The annotation of *C. melo *scaffoldMRGH63 and scaffold00077 was complemented using information from *c*a. 57 kb and 96 kb, respectively, of unpublished melon sequence (represented in the figure as red edge boxes). Figure drawn to scale.

Four of the longest scaffolds (9, 15, 77 and MRGH63, totalling 782 kb) were used to search the cucumber genome assembly [50] for syntenic regions, as described in the Methods section. As it can be expected from the close phylogenetic relatedness of these two species, a high degree of collinearity was found in all four regions analysed (Figure 2). The relative syntenic quality (see the Methods section) ranged from 84% (for scaffold MRGH63) to 97% (for scaffold00015), averaging 92%, and the homologous protein sequences rendered in all cases e-values lower than 1E-46 with an average identity of 87% when aligned using BLASTP (see Additional file 4 Table S3). Regarding the annotation characteristics of the predicted genes, the average protein lengths of the four melon regions analyzed were x0.8-x1.2 those of cucumber, with the syntenic melon genes being, as an average, only x0.96 smaller than the cucumber ones; the average number of exons of the melon syntenic regions were x0.84-x1.1 those of the cucumber regions, with the syntenic melon genes having, as an average, only x0.92 less exons than the cucumber ones; also, although the average exon length of all syntenic melon genes was almost identical to that of cucumber, the average intron length of the syntenic melon genes was x1.3 that of their cucumber counterparts (Additional file 4 Table S3).

Besides, the orientation of the putative syntenic genes was found to be conserved in all cases. However, a number of genes were duplicated in melon. These included the expansion of a cluster of NBS-LRR genes present in scaffold MRGH63, which is particularly interesting as the *Vat *gene and other disease resistance genes have been mapped to this region [33]. NBS-LRR genes are the main family of resistance genes in plants, and are frequently found in clusters [51]. Highly conserved gene order and content together with 95% of sequence similarity over coding regions has already been reported by Huang *et al*. based on the comparison of four sequenced BAC clones against the sequenced cucumber genome [7].

Besides the duplication of several genes, a major difference between cucumber and melon syntenic regions is the number of transposon insertions [Figure 2]. The cucumber sequences analysed contain only two retrotransposon insertions, one of which seems very old as it is highly degenerated. On the contrary, the melon syntenic regions contain three DNA transposons (two hATs and one MULE) and 15 retrotransposons (most of them from the *Gypsy *superfamily), including the degenerated retrotransposon found in cucumber. In particular, transposon activity appears to account for the expansion of *ca*. 60 kb in the melon scaffold0077 relative to its cucumber counterpart. In scaffold MRGH63, a localised transposon number amplification together with duplication of melon resistance gene homologs (see below) accounts for an 88 kb-long expansion of the sequence of melon relative to that of cucumber. Also, transposons were found to be putatively involved in gene disruption processes in scaffolds 9 and MRGH63.

These results suggest that transposition activity after the divergence of the two ancestors of melon and cucumber has been low in cucumber but very high in melon. This transposon amplification and mobilization could be a reason for the 1.8× increase in size of the melon syntenic regions. Bearing in mind that the melon genome is estimated to be 1.3× greater that of cucumber, it can tentatively be assumed that transposon activity may be mainly responsible for that difference in genome sizes.

It is interesting to note that almost half of the melon specific transposons are interspersed with NBS-LRR predicted genes that potentially form resistance gene clusters. Gene duplications and transposon insertions have been proposed to provide a structural environment that permits unequal crossovers and interlocus gene conversion allowing rapid evolution of resistance genes [51]. In addition, the presence of active retrotransposons interspersed with resistance genes may also contribute to the resistance gene regulation by silencing related mechanisms [52]. A detailed analysis of syntenic regions containing putative resistance genes between melon and cucumber may provide new information on the evolution of resistance genes and the development of new resistances in cultivated crops.

## Conclusion

A set of 57 BAC clones from a double haploid line of melon was sequenced in two pools with the 454 system using both shotgun and paired-end approaches followed by bioinformatic assembly of the fragments obtained. From this assembly it was possible to obtain most likely complete sequences for 50 of these BACs, as judged by the length and the presence of BAC-end sequences, with a final coverage of 39×. The accuracy of the assembly was excellent, compared with a BAC clone already sequenced with the Sanger method, except in a small number of repetitive sequences, consistent with other 454 sequencing projects [37,38]. These results show that 454-sequencing of pooled BACs, using both shotgun and paired-end libraries, is a feasible strategy for sequencing long stretches of genomic sequence from medium-size genomes such as that of melon. However, correction using other sequencing techniques would be needed for medium to high repetitive content regions.

The analysis of the fraction (around 1.5%) of the melon genome obtained provides a pilot overview of this species' genomic structure. Predicted gene annotations were confirmed in 73% of the cases by comparison with EST collections. This is probably a good measure of the completeness of the transcriptome information currently available for this species. The analysis of the sequences provides an interesting overview of the features such as microsatellite content, gene density and average protein length, revealing similarity to that of its close relative, cucumber.

Finally, the comparison of four melon regions totalling 782 kb against the genomic sequence of cucumber (the only other Cucurbit species where a draft genome sequence is available) reveals a high degree of collinearity between both species. The analysis of the detected syntenic regions suggests that the size difference of the two genomes is due to the expansion of intergenic regions, mainly through the activity of transposable elements in melon after the divergence of the two species. It is particularly interesting to note that almost half of the detected melon-specific transposons are interspersed with NBS-LRR predicted genes that potentially form resistance gene clusters. We have confirmed the utility of this sequencing method for small genomic fractions, and the analysis of the data thus obtained has expanded our understanding of the melon genome structure and the mechanisms underlying its evolution.

## Methods

### BAC library

A *Bam*HI BAC library from the double-haploid melon line 'PIT92' (PI 161375 × T111) was previously developed in our laboratory using pECBAC1 as cloning vector [[12], http://hbz7.tamu.edu/homelinks/bac_est/vector/sequence/sequence.htm]. With 23,040 BAC clones distributed in sixty 384-well plates, an average insert size of 139 kb and 20% empty clones, the library represents 5.7 genomic equivalents of the haploid melon genome.

### DNA extraction

Two pools of 35 and 23 BACs were selected for the analysis. Individual preinocules were grown on 1 ml 1 × LB plus 12.5 μg/ml chloramphenicol at 300 rpm, 37°C, for 17 h. The following day, 30 μl of each BAC clone from the preinocules were added into 50 ml tubes containing 20 ml 1 × LB plus 12.5 μg/ml chloramphenicol, and grown at 37°C, 300 rpm for 15 h. The grown cultures were then mixed to produce two separate volumes representing the two BAC pools and the bacterial cells were harvested by centrifugation at 6,000 × g for 15 min at 4°C.

Genomic DNA-free BAC DNA extraction was performed using the QIAGEN^® ^Large-Construct Kit (Cat. No. 12462) following the manufacturer's instructions. Both final DNA pellets were resuspended in 500 μl TE pH 8.0 each.

### DNA sequencing

All sequencing was performed with a Roche 454 Genome Sequencer machine using FLX chemistry. Two DNA extractions were done from the 35-BACs pool, one to create a shotgun library and the other one to create a 3 kb paired-end library. The shotgun library was used for one titration run and one full run performed by Lifequencing S. L. at their premises in Valencia, Spain. The paired-end library was sequenced on two quarters of a plate followed by a full run performed at our 454 sequencing facility. For the 23-BACs pool, one DNA extraction was done which served to create a shotgun and a 3 kb paired-end library. The shotgun library was sequenced with a full run while the paired-end library was sequenced on three eighths of a plate; both runs were performed at our 454 sequencing facility.

### Sequence assembly

Sequence assembly was done using Newbler version 2.3 with default parameters. Reads from all BACs were processed together in one assembly run. The sequence of *E. coli *strain DH10B (NC 010473.1) was used as screening database and the vector pECBAC1 as trimming database, but without 30 bp of sequence flanking either side of the BamHI restriction site (see below) http://hbz7.tamu.edu/homelinks/bac_est/vector/sequence/sequence.htm. Additional assemblies of each BAC pool were independently done using Newbler versions 2.3 and 2.0 (data now shown); results of these assemblies served for comparison purposes and only in some cases helped to manually correct some scaffolds in the global assembly.

Sequences of the genetic markers previously anchored to the analyzed BACs as well as some BAC-end sequences previously available in our laboratories (GenBank Acc. Nos. can be found in the Additional file 3 Table S2) were used to assign a sequence to a specific BAC. Based on this information, in some cases we could join two scaffolds that corresponded to the same BAC into a single superscaffold that would represent the whole BAC insert. In these cases a gap was introduced between the scaffolds so that the final sequence had the size of the average insert size of the BAC library. The manually introduced gaps accounted for 7.25% of all the gaps in the assembly. The sizes of these gaps in nucleotides are as follow: 500 in Scaffold52B05; 1,209 in Scaffold45K01; 1,538 in Scaffold11I12; 1,831 in Scaffold54E01; 2,288 in Scaffold55F19; 2,586 in Scaffold59B11; and 12,064 in Scaffold54J04.

In order to study how many of the assembled contigs and scaffolds represented the complete sequence of BACs, those sequences were searched for BAC borders in the following ways: 1) by searching at their extremes the 30 bp sequence corresponding to pECBAC1; 2) by blasting against individual reads containing the 30 bp sequence and 3) by blasting against BAC-end sequences that were already available for some of the sequenced BACs [see Additional File 3 Table S2].

### Sequence annotation

*Ab initio *gene prediction was performed using the command-line version of Augustus 2.3 software http://augustus.gobics.de/ using *A. thaliana *as plant model. The melon unigene v3 collection at ICUGI [11] was used to improve the Augustus prediction, setting the minimum identity parameter to 92. In some cases, the FGENESH annotation software at http://linux1.softberry.com/berry.phtml, with *Arabidopsis *as plant model, was used to complement or improve the Augustus annotation. The predicted coding sequences were checked against the non-redundant protein databases at NCBI using BLASTP searching for protein homologs.

tRNA genes were predicted using the tRNAscan-SE 1.21 software http://lowelab.ucsc.edu/tRNAscan-SE/ and rRNA genes were identified with RNAmmer 1.2 server http://www.cbs.dtu.dk/services/RNAmmer/. Simple sequence repeats (SSRs) were searched for using the msatcommander 0.8.2 software http://code.google.com/p/msatcommander/; the minimum repeat lengths considered were: 10 bp (mononucleotides), 12 bp (di- and trinucleotides), 16 bp (tetranucleotides), 20 bp (pentanucleotides) and 24 bp (hexanucleotides).

Transposons were annotated using *Ab initio *and sequence similarity searches integrated in a pipeline based on Dawgpaws [53]. The programs used for *de novo *prediction of LTR retrotransposons included LTR_STRUC [54], LTR_finder [55] and LTR_seq [56], and vary in the type of structures they look for, their stringency and their search algorithms. The homology-based approach consisted of searching for sequences that show a high degree of similarity to known transposons. For this, we compiled nucleotide databases of already characterized transposons obtained from the RepBase database [57] as well as NCBI [58]. Likewise, we constructed protein sequence databases of transposases from various transposon families, searching NCBI for combinations of keywords such as "transposase" and "CACTA", "hAT", "Mariner", "Mutator" or "PIF". This approach is useful for corroborating results obtained from the *de novo *programs, as well as identifying other types of transposons such as DNA transposons. The output of these various programs was converted into gff3 format and uploaded into the Apollo genome viewer and annotation tool [59], along with the gene annotations, for manual curation. As a first step, each scaffold was examined and putative transposons were identified according to the computational evidence. These were then manually inspected to look for LTRs or TIRs, query NCBI to determine which family they belong to, and resolve instances of nested or truncated elements. These *bona fide *transposons were used to query the set of scaffolds in similarity searches, aiming at identifying partial or degenerated copies and defining transposon families. This third step is particularly relevant when a large amount of sequence data is available, as aligning many copies of an element aids to precisely define its borders and find consensus sequences. At this point, with the current fraction of the genome available, we have not found enough copies of a single element to perform this part of the analysis.

### Synteny analysis

Four annotated melon scaffolds were analysed for homology with the *Cucumis sativus *genome assembly deposited in Phytozome v5 [50], using the BLASTN algorithm. The selected cucumber regions were annotated the same way as the melon BACs. Pairs of homologous genes were tentatively selected on the basis of the gene annotation and then confirmed by performing BLASTP alignments of the correspondent predicted proteins. Syntenic regions were defined as contiguous regions containing two or more homologous genes in *C. melo *and *C. sativus*, irrespective of orientation and exact order of genes, based on the results of BLASTP comparisons. The relative syntenic quality in a region, expressed as a percentage, was calculated by dividing the sum of the conserved genes in both syntenic regions by the sum of the total number of genes in both regions, excluding transposable elements and collapsing tandem duplications [60].

## Authors' contributions

VMG conducted BAC DNA extractions, helped to manually correct the final sequence assembly, annotated scaffolds, and drafted the manuscript, AB led the pre-processing of the sequence raw data, produced the sequence assemblies, and helped drafting the manuscript, EMH and JMC were in charge of the transposon content analysis and helped drafting the manuscript, GM constructed the 23 BAC pool shotgun and all paired-end libraries and performed the sequencing reactions, JGM participated in the project design, coordinated the BAC sequencing, participated in the discussion of results, and helped to draft the manuscript, PP conceived and coordinated the project, and helped to draft the manuscript. All authors read and approved the final manuscript.

## Note

The C. melo BAC nucleotide sequences are available in the DDBJ/EMBL/GenBank databases under the accession numbers HM854749-HM854824. The raw data can be found in the SRA archive of the NCBI under the accession number SRA024701.1.

## Supplementary Material

Additional file 1 **Figure S1**. Schematic representation of the MRGH63 contig. Click here for file

Additional file 2 **Table S1**. Genetic markers anchored to the sequenced BAC clones. Click here for file

Additional file 3 **Table S2**. Sequence and annotation characteristics of the assembled scaffolds and contigs. Click here for file

Additional file 4 **Table S3**. Annotation characteristics of the *C. melo *and *C. sativus *syntenic regions Click here for file
