# Supplementary material for: Sequencing of 6.7 Mb of the melon genome using a BAC pooling strategy
Source: BMC Plant Biol. 2010 Nov 12;10:246. doi: 10.1186/1471-2229-10-246 (PMC3095328; doi:10.1186/1471-2229-10-246)
Supplement: Additional file 1 — Figure S1. Schematic representation of the MRGH63 contig. [file 1471-2229-10-246-S1.PDF]

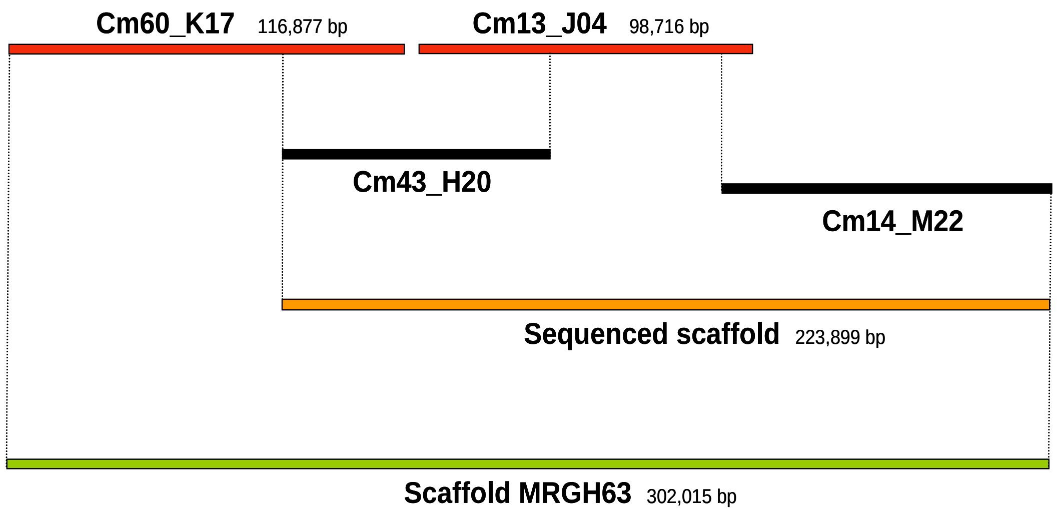

Figure S1

Schematic representation of the MRGH63 contig. The contig consists of two previously sequenced BAC clones (Cm60_K17, Acc. No. AF499727, and Cm13_J04, Acc. No. EF657230) and two additional clones (Cm43_H20 and Cm14_M22). BAC clones Cm13_J4, Cm43_H20 and Cm14_M22 were among those pooled and 454-sequenced. The contig sequence (‘scaffold MRGH63’) includes 78,116 bp from the known Cm60_K17 sequence.
